# Patent Foramen Ovale, Ischemic Stroke and Migraine: Systematic Review and Stratified Meta-Analysis of Association Studies

**DOI:** 10.1159/000341924

**Published:** 2012-10-11

**Authors:** Daniel Davis, John Gregson, Peter Willeit, Blossom Stephan, Rustam Al-Shahi Salman, Carol Brayne

**Affiliations:** ^a^Department of Public Health and Primary Care, University of Cambridge, Cambridge; ^b^Division of Clinical Neurosciences, University of Edinburgh, Edinburgh, UK; ^c^Department of Neurology, Innsbruck Medical University, Innsbruck, Austria

**Keywords:** Systematic review, Meta-analysis, Patent foramen ovale, Cryptogenic stroke, Migraine

## Abstract

**Background:**

Observational data have reported associations between patent foramen ovale (PFO), cryptogenic stroke and migraine. However, randomized trials of PFO closure do not demonstrate a clear benefit either because the underlying association is weaker than previously suggested or because the trials were underpowered. In order to resolve the apparent discrepancy between observational data and randomized trials, we investigated associations between (1) migraine and ischemic stroke, (2) PFO and ischemic stroke, and (3) PFO and migraine.

**Methods:**

Eligibility criteria were consistent; including all studies with specifically defined exposures and outcomes unrestricted by language. We focused on studies at lowest risk of bias by stratifying analyses based on methodological design and quantified associations using fixed-effects meta-analysis models.

**Results:**

We included 37 studies of 7,686 identified. Compared to reports in the literature as a whole, studies with population-based comparators showed weaker associations between migraine with aura and cryptogenic ischemic stroke in younger women (OR 1.4; 95% CI 0.9–2.0; 1 study), PFO and ischemic stroke (HR 1.6; 95 CI 1.0–2.5; 2 studies; OR 1.3; 95% CI 0.9–1.9; 3 studies), or PFO and migraine (OR 1.0; 95% CI 0.6–1.6; 1 study). It was not possible to look for interactions or effect modifiers. These results are limited by sources of bias within individual studies.

**Conclusions:**

The overall pairwise associations between PFO, cryptogenic ischemic stroke and migraine do not strongly suggest a causal role for PFO. Ongoing randomized trials of PFO closure may need larger numbers of participants to detect an overall beneficial effect.

## Introduction

Modifiable risk factors for ischemic stroke may be targets for primary or secondary prevention, especially if the strength of their association suggests that they might be causal. Up to one third of ischemic strokes are ‘cryptogenic’ because a recognized cause is not identified [1]. One putative cause of cryptogenic ischemic stroke is paradoxical embolism through patent foramen ovale (PFO) [2], itself a consequence of failure of the septa on either side of the fetal interatrial shunt to fuse after birth. Moreover, suggested associations between PFO and migraine are of interest because migraine has also been considered a risk factor for ischemic stroke [3].

Non-blinded and non-randomized interventional studies of percutaneous PFO closure have suggested benefits of PFO closure on recurrent cryptogenic ischemic stroke [4,5,6] and refractory migraine [7,8,9,10], but randomized controlled trials have not confirmed these effects [11,12]. This suggests the possibility that the reported observational data, like the uncontrolled trials, may have been biased or confounded. While the randomized trials may have been underpowered to detect a benefit of closure, this may have been driven by the original estimated effect size being based on a stronger association than is actually the case.

In this context, it is timely to review the observational studies and the way in which previous meta-analyses addressed methodological biases when pooling estimates of association. To date, systematic reviews have suggested significant associations between migraine and ischemic stroke [odds ratio (OR) 2.2; 95% confidence intervals (CI) 1.6–3.0] [13], PFO and ischemic stroke (OR 2.9; 95% CI 2.1–4.0) [14], and PFO and migraine (OR 2.5; 95% CI 2.0–3.1) [15]. However, the degree of bias in observational data differs between studies, and not accounting for these differences may lead to propagation of these biases in the pooled estimates [16]. One approach to this issue is to stratify analyses based on methodological design, thereby reducing heterogeneity in observational studies in the meta-analysis.

**Fig. 1:**
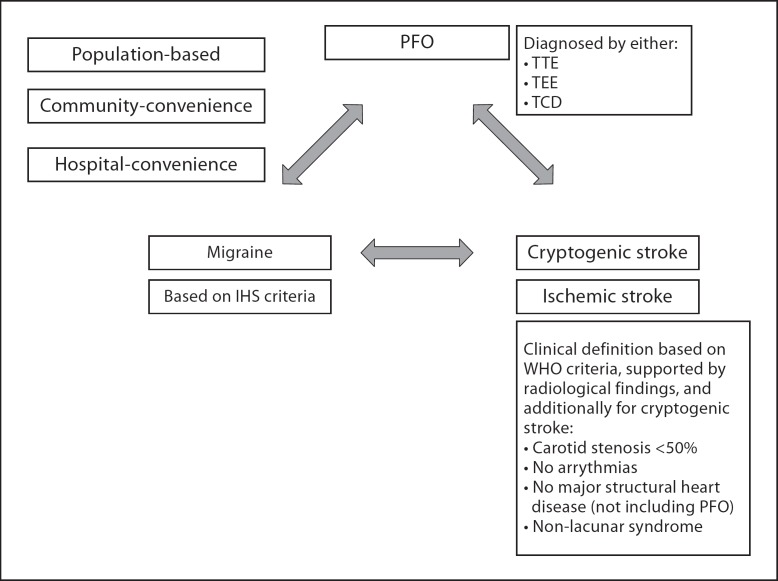
Diagramatic representation of systematically examined associations. Summary of relationships between pairwise associations, outlining operationalized definitions of exposures and outcomes.

Here, we set out to refine the interpretation of the current literature in order to (1) quantify the interrelated associations between migraine, PFO and stroke, and (2) to inform the design of randomized trials of PFO closure. Therefore, we undertook a series of systematic reviews and meta-analyses investigating the pairwise associations between a past history of migraine, PFO and the occurrence of cryptogenic ischemic stroke or all ischemic stroke (fig. 1), applying consistent eligibility criteria across all three relationships. We specifically gave priority to population-based studies, prospective cohort designs and studies that had matched or adjusted for known confounders for stroke, with a view to stratifying by study design and restricting meta-analyses to methodologically similar population sampling frames.

## Methods

The protocol followed the Meta-analysis of Observational Studies in Epidemiology (MOOSE) guidelines [17] (online suppl. checklist; for all online supplementary material, see www.karger.com?doi=10.1159/000341924; study characteristics of all included studies are given in full, along with referenced PRISMA flowchart detailing excluded studies).

### Eligibility Criteria

We searched for cohort, case-control or cross-sectional studies unrestricted by demographic features or language of publication, which had been conducted in population or hospital settings, and which compared the associations between any two relationships (fig. 1). We required studies defining ischemic stroke by WHO criteria (supported by radiological findings) and cryptogenic ischemic strokes defined as non-lacunar clinical syndromes without carotid stenosis >50%, cardiac arrhythmias or structural heart disease (excluding PFO). To be included, we required studies that did not report an outcome of cryptogenic ischemic stroke but instead report total first-ever or recurrent ischemic stroke with adjustment for known risk factors for ischemic stroke (specifically hypertension, diabetes, hypercholesterolemia, atrial fibrillation, smoking, alcohol, family history). We included studies defining migraine according to the International Headache Society (IHS) classification [18,19]. PFO must have been diagnosed by contrast transthoracic echocardiogram (TTE), transesophageal echocardiogram (TEE) or transcranial Doppler (TCD) ultrasound during Valsalva maneuver. Cerebral infarction in the context of complex migraine is a well-recognized clinical phenomenon, and so studies reporting migrainous infarction were not considered further. We did not include data from placebo arms of randomized trials because interventional studies are not required to use a consistent sampling frame. We excluded studies of children, pregnant women and those reporting associations within family pedigrees.

### Information Sources

D.D. searched Medline from 1950 on PubMed, Embase from 1980 on NHS Evidence Health Information Resources, and Science Citation Index on Web of Science from 1950, all to April 2012. We sought journal publications as well as conference abstracts and contacted authors of abstracts to determine whether further data had been published. In addition, the bibliographies of included articles and other systematic reviews were screened.

### Search Terms and Study Selection

D.D. used comprehensive textword and Medical Subject Heading (MeSH) to find relevant studies in humans (full search terms given in MOOSE checklist, online suppl. material). Four reviewers (D.D., J.G., P.W., B.S.) independently screened articles by title and abstract after de-duplication through first author surname, title and first page number with those for full-text review, and subsequent inclusion agreed by consensus. We sought every publication relating to each included study using a search strategy based on the full name of the study or its acronym, as well as forward searching using citation index databases.

### Data Extraction

Two reviewers independently extracted study data using standardized forms developed after piloting and critically appraised each study using the Newcastle-Ottawa scale (NOS) [20]. The NOS assesses population selection, comparability of cases and controls, and adequacy of outcome assessment (including outcome ascertainment and attrition). In keeping with the MOOSE guidelines, we did not use the NOS to determine study inclusion but to guide the classification of studies by similarities in design.

We classified a hierarchy of population sampling methods from lowest to highest risk of bias (online suppl. table S1), where ‘population-based’ refers to a sampling frame including all subgroups within a geographical population, unrestricted by demographic or clinical features, ‘community-convenience’ indicates selection from outpatient participants, and ‘hospital-convenience’ denotes participants recruited only from hospital inpatients. We considered cryptogenic ischemic stroke and ischemic stroke as separate outcomes.

### Data Analysis

J.G. and P.W. conducted the statistical analyses using STATA version 10.1. Where multiple estimates of association were reported, the most adjusted estimate was used in analyses. Summary statistics for hazard ratios (HR), relative risks (RR) or OR and their accompanying 95% CI were calculated using a Mantel-Haenszel fixed-effect model. Statistical heterogeneity was assessed with the I^2^ statistic.

## Results

We identified 7,686 studies (online suppl. MOOSE checklist). We reviewed the full text of 116 studies, and excluded studies are fully referenced in the PRISMA flow diagrams (online suppl. fig. S1–S3). Supplementary table S2 gives further details of any study considered in any previous systematic reviews and meta-analyses, if any such study was excluded here. Finally, we included 37 studies in this review. We have separately summarized study characteristics, their results (and their meta-analysis, where appropriate) and risk of bias according to the association being investigated. Full details of all reviewed studies are given in online supplementary tables S3–S5. Table 1 shows the characteristics of only those included studies with either population-based sampling frames or a prospective design.

### Migraine and Ischemic Stroke

**Fig. 2:**
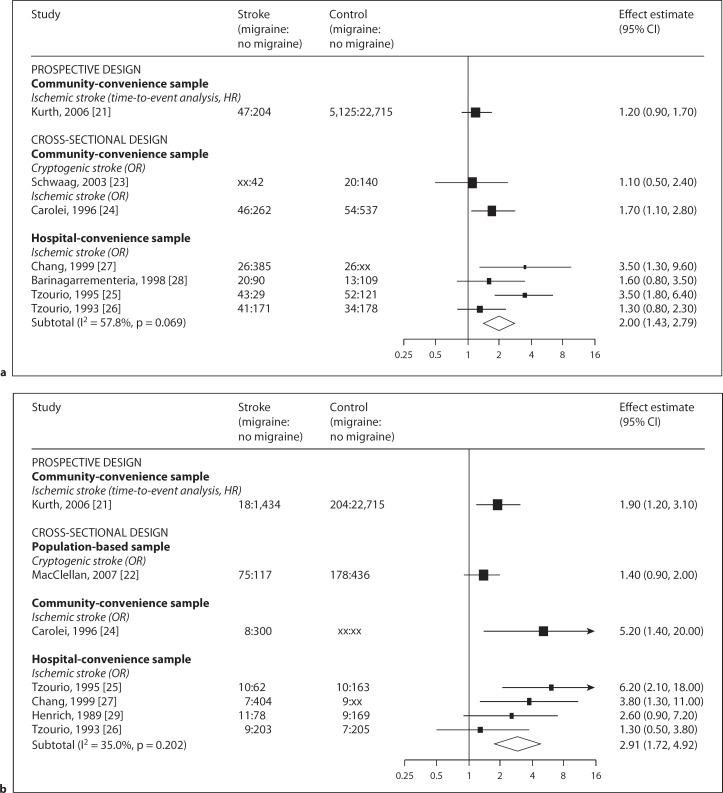
**a** Meta-analysis of the reported associations between migraine and ischemic stroke (by study design and sampling frame). **b** Meta-analysis of the reported associations between migraine with aura and ischemic stroke (by study design and sampling frame). Forest plot showing results of meta-analysis. Studies are grouped by design and sampling frame. Black squares are proportional to the study size, lines represent 95% confidence intervals. The open diamond summarizes the pooled estimate within each stratum. Absolute values are given for cases and controls (numerator and denominator), xx indicates where these were not reported.

We identified one prospective and one population-based case-control study of women with migraine with aura with conflicting results (fig. 2).

#### Prospective Studies

The Women's Health Study (WHS), which included 27,840 female health care professionals aged >45 years, was the only prospective study in this section to meet all inclusion criteria [21]. The most fully adjusted model estimated an association with ischemic stroke (HR 1.2; 95% CI 0.9–1.7 for migraine and HR 1.9; 95% CI 1.2–3.1 for active migraine with aura).

#### Population-Based Case-Control Studies

One case-control study used a population-based sampling frame [22]. In women aged 15–49 years, 192 participants with migraine with aura were not associated with cryptogenic stroke (adjusted OR 1.4; 95% CI 0.9–2.0). In a small subgroup of these women with known PFO, there was no evidence of a different association (adjusted OR 2.1; 95% CI 0.8–5.3; n = 21) compared to the group known to not have PFO (adjusted OR 1.5; 95% CI 1.0–2.2; n = 142).

#### Non-Population-Based Case-Control Studies

Figure 4a shows the estimated associations for any history of migraine and ischemic stroke, stratified by study design. The reported association of one study comparing cryptogenic stroke cases with community-convenience controls (OR 1.1; 95% CI 0.5–2.4) [23] was somewhat weaker than the association in a study comparing ischemic stroke cases [24] with community-convenience controls (OR 1.7; 95% CI 1.1–2.8) than the pooled risk estimate of studies of ischemic stroke with hospital-convenience controls [25,26,27,28] (pooled OR 2.0; 95% CI 1.4–2.8; I^2^ = 58%). Figure 2b shows the meta-analysis of studies reporting migraine with aura subtypes [25,26,27,29]. This yielded a pooled OR of 2.9 and 95% CI of 1.7–4.9 (I^2^ = 35%). Failure to account for atrial fibrillation was a feature common to all these studies.

### PFO and Ischemic Stroke

**Fig. 3:**
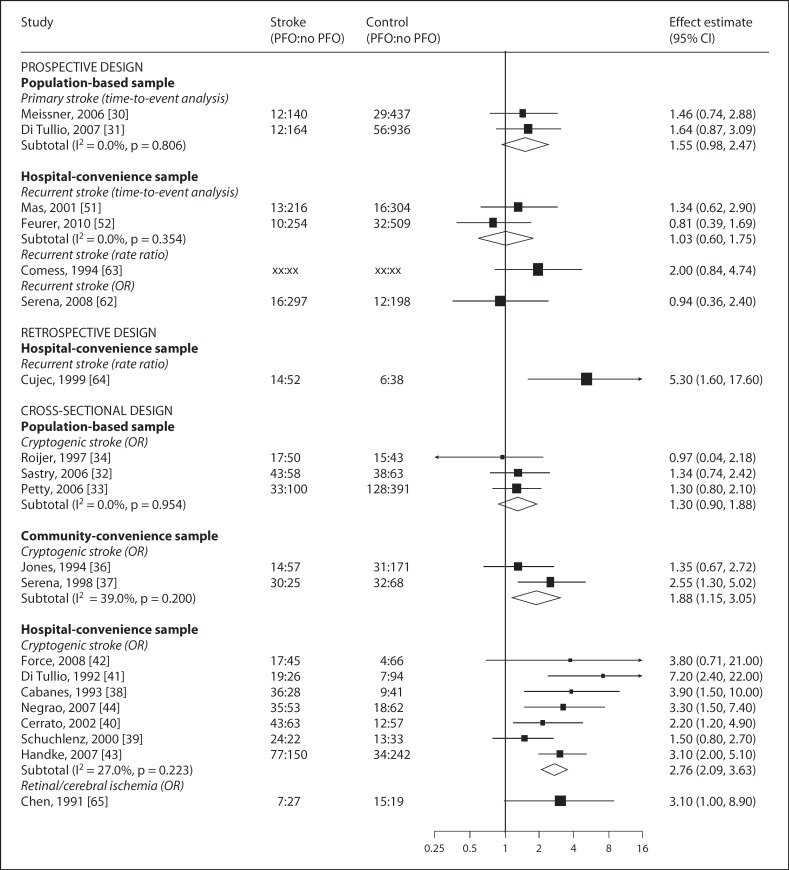
Meta-analysis of the reported associations between PFO and ischemic stroke (by study design and sampling frame). Forest plot showing results of meta-analysis. Studies are grouped by design and sampling frame. Black squares are proportional to the study size, lines represent 95% confidence intervals. The open diamond summarizes the pooled estimate within each stratum. Absolute values are given for cases and controls (numerator and denominator), xx indicates where these were not reported.

Pooled results from prospective and population-based studies did not demonstrate an association between PFO and ischemic stroke (fig. 3).

#### Population-Based Prospective Studies

We identified 2 population-based cohort studies in essentially stroke-free populations where ischemic stroke adjusted for known vascular risk factors was the main outcome (fig. 3) [30,31]. The pooled HR for risk of primary ischemic stroke in relation to PFO was 1.6 with 95% CI of 1.0–2.5 (I^2^ = 0%). The other prospective studies reported no statistically significant association (where p < 0.05), either individually or if pooled (OR 1.0; 95% CI 0.6–1.8; I^2^ = 0%) (fig. 3).

#### Population-Based Case-Control Studies

Three studies compared cryptogenic ischemic stroke with healthy controls sampled from the general population, yielding a pooled OR of 1.3 and 95% CI of 0.9–1.8 (I^2^ = 0%) (fig. 3) [32,33,34]. Two studies performed subgroup analyses to assess the association of larger shunts with stroke [32,33]. Pooling the association across studies yielded an OR of 1.3 and 95% CI of 0.8–2.1 (I^2^ = 79%).

#### Non-Population-Based Case-Control and Cross-Sectional Studies

A total of 10 studies compared cryptogenic ischemic stroke with: (1) healthy community-convenience controls [35,36]; (2) controls undergoing echocardiography for non-stroke indications [37,38,39], or (3) ischemic stroke of known cause, adjusted for other vascular risk factors [40,41,42,43,44]. When subdivided by a population sampling frame, the pooled ORs for community-convenience and hospital-convenience controls were 1.9 (95% CI 1.2–3.1; I^2^ = 39%) and 2.8 (95% CI 2.1–3.6; I^2^ = 27%), respectively (fig. 3).

### PFO and Migraine

**Fig. 4:**
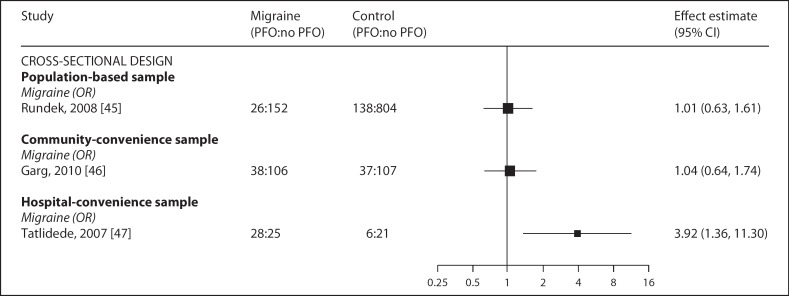
Meta-analysis of the reported associations between PFO and migraine (by study design and sampling frame). Forest plot showing results of meta-analysis. Studies are grouped by design and sampling frame. Black squares are proportional to the study size, lines represent 95% confidence intervals.

We found one population-based study, and this did not show an association between PFO and migraine (fig. 4).

#### Cross-Sectional and Case-Control Studies

In addition to the stroke outcomes above, the population-based NOMAS study [45] reported the association between PFO and migraine as OR of 1.0 (95% CI 0.6–1.6), and this was not different when only considering migraine with aura (OR 1.0; 95% CI 0.7–1.7). Garg et al. [46] reported no association with PFO (OR 1.0; 95% CI 0.6–1.7) in 144 stroke-free migraineurs recruited from a tertiary clinic who were compared to age- and sex-matched controls selected from a register of healthy volunteers. The other case-control study used convenience controls, and the estimate for any history of migraine and PFO was OR of 3.9 (95% CI 1.4–11) (fig. 4) [47]. Two further abstracts were identified that reported population-based associations, and full publication of both studies is awaited [48,49].

## Discussion

### Summary of Evidence

For each pairwise association between migraine, PFO and ischemic stroke, there is inconsistent evidence, and the strength of the reported associations is dependent on study design. Overall, associations apparent in case-control studies were not evident in the more generalizable population-based studies with sufficiently valid assessments of exposure and outcome.

#### Migraine and Ischemic Stroke

In the subset of younger women with migraine with aura and cryptogenic stroke, there is no clear association [22], and there are no studies with a strict population-based sampling frame describing this relationship in men (of any age). However, there is some evidence from a prospective study suggesting an association in female health workers aged >45 years with migraine with aura and ischemic stroke [21], though atrial fibrillation was not accounted for despite a separate report demonstrating an association between this and stroke [50].

#### PFO and Ischemic Stroke

Cohort studies did not demonstrate an association between PFO and first-ever [30,31] or recurrent ischemic stroke risk [51,52], findings which are supported by three case-control studies comparing PFO in cryptogenic ischemic stroke with population-based controls [32,33,34].

#### PFO and Migraine

The only population-based study investigating the association between PFO and migraine with or without aura was cross-sectional and found no association [45].

### Strengths and Limitations

This is the first review on this topic to stratify analyses on methodological grounds, which may explain why our conclusions differ from earlier meta-analyses. In applying a consistent approach to article selection and critical appraisal, we have been able to assess the evidence systematically across the three relationships. Our approach to meta-analysis has had the advantage of yielding low statistical measures of heterogeneity (I^2^). While there are other procedures for addressing heterogeneity [53], our method has sound face validity. By identifying the observational studies at least risk of bias, we base our conclusions on the most epidemiologically applicable data.

For studies of migraine and ischemic stroke, there was inconsistency in controlling for possible confounders, in particular atrial fibrillation. Accordingly, the one prospective study to report a positive association (the WHS) should be considered with caution in this light.

The two population-based prospective studies of PFO and first-ever stroke risk had very similar results and did not demonstrate an association. It is possible that longer follow-up might have detected a small contribution of PFO to ischemic stroke risk. However, given the age-related incidence of more well-recognized risk factors for ischemic stroke, the power to detect an attributable risk from PFO probably diminishes with time. The population-based case-control studies were in agreement, though the findings from Roijer et al. [34] are problematic because the age matching was unsuccessful, with controls being younger. In addition, although ‘major potential cardio-embolic sources’ were excluded, a higher frequency of atrial fibrillation was reported in the cases. Despite these possible biases, the overall conclusions are acceptable in the context of the other population-based studies.

The only population-based data on the association between migraine and PFO assessed migraine in those reporting a past history of headache during a screening interview. Given the mean age of the participants (69 years), it has been suggested that recall bias may have caused an underestimation of migraine history, though the reported prevalence of migraine is consistent with other estimates from the general population [45]. Finally, because the pooled results from population-based studies did not demonstrate any clear associations, it was not possible to examine for any potential interactions or effect modifiers.

There are three main reasons that population-based observational studies may consistently report no association between migraine, PFO and cryptogenic stroke. Firstly, any misclassification of exposure status may bias any true association towards the null. In particular, studies employing TTE may have not detected PFO as reliably as more sensitive methods. Secondly, population-based studies may themselves been underpowered to detect the population-attributable fraction for stroke from PFO. Thirdly, the null hypothesis that PFO is not associated with stroke or migraine in unselected populations cannot be rejected. Therefore, there are general implications for trials considering PFO closure in terms of how to assess PFO, which subpopulations to sample and the sample size required to demonstrate a modifiable effect.

### Relationship to Other Meta-Analyses

Previous meta-analyses have separately examined the relationships reviewed here. With regard to migraine and ischemic stroke, two recent systematic reviews have been conducted [13,54]. The eligibility criteria in these reviews did not specifically focus on cryptogenic stroke and included a larger number of studies. One review pooled – with equal weighting – evidence across cohort (HR) and case-control studies (OR), finding an association measure of 2.2 (95% CI 1.5–3.0) [13]. The other meta-analysis adopted similar methods but included a separate analysis restricted to studies at low risk of bias as determined by the authors (fewer than three ‘poor’ scores in different areas of the study design), in essence assigning an overall score for quality [54]. However, even in the analysis restricted to high-quality studies, the statistical heterogeneity was still sufficiently high to require a random-effects meta-analysis (I^2^ = 68%). Because this in effect combines studies across different populations, it is no longer clear to which population the pooled estimate then applies. Assigning an overall quality score does not in itself address the biases within individual studies. We avoided this by stratifying our analyses where risk of bias due to study design and population sampling is likely to be similar across studies.

Two systematic reviews on the relationship between PFO and cryptogenic ischemic stroke have been published [14,55]. Both of these only examined case-control studies, thus do not account for evidence derived from prospective studies, and their results are consequently less conservative than ours, where the PFO-stroke association was estimated as OR of 3.0 (95% CI 2.0–4.3) for studies of PFO in cryptogenic stroke compared to non-stroke controls [55]. For the PFO-migraine association, another meta-analysis demonstrated an OR of 2.5 (95% CI 2.0–3.1; PFO in migraineurs) and an OR of 5.1 (95% CI 4.7–5.6; migraine in PFO patients) [15], though the systematic review predated the NOMAS report and also acknowledged that publication bias was likely. Two abstracts await full publication but appear to report associations in population-based samples. The Genetic Epidemiology of Migraine (GEM) study found migraine and PFO to be related only where PFO was also associated with an atrial septal aneurysm [48]. CAMERA is a MRI substudy of GEM, reporting a significant association in migraine with aura and PFO (OR 2.1; 95% CI 1.1–3.9) [49]. However, it is unlikely that pooling the preliminary result with the NOMAS data would substantially alter these conclusions given that NOMAS is a much larger study.

### Implications for Randomized Trials

The findings of this systematic review have implications for trials of PFO closure. If the underlying association is weaker than previously recognized (or non-existent), detecting a treatment effect may require enormous sample sizes (or may be impossible). However, if randomized trials demonstrate a treatment effect, this would provide evidence not only for an association between PFO and ischemic stroke or migraine, but one that is causal.

Our findings may account for the results of the CLOSURE trial [12]. The trial had an event rate (TIA and stroke) of 6% in the control arm and 2% in the closure arm. Also considering adverse effects of treatment, there were 7.7 and 5.9% events in the control and intervention groups, respectively (p = 0.30). Regardless of the concerns surrounding peri-procedural atrial fibrillation in this and other cohorts [56], one interpretation of the trial is that the underlying association is too small to be modified by an intervention with this efficacy.

The Migraine Intervention With STARFlex Technology (MIST) trial is the only double-blinded sham-controlled intervention trial in patients with refractory migraine with aura [11]. Though questions remain in respect of the possibility that interatrial shunts were not completely closed [57,58], the reported data suggest no benefit of PFO closure on migraine. It has been proposed that MIST participants were not representative of those in whom treatment benefit was observed in uncontrolled trials (i.e. those migraineurs spontaneously improving after PFO closure for cryptogenic stroke) [59]. This observation is supported by the community-based studies finding no association between migraine and PFO in stroke-free populations identified in this review.

## Conclusions

Our findings do not support an association between PFO in patients with cryptogenic ischemic stroke. Because the weak association between PFO and ischemic stroke in methodologically rigorous studies questions the causal role of PFO, randomized controlled trials of PFO closure constitute an essential test of the causal relationship. However, the weakness of the association in observational epidemiological studies, as well as the effectiveness of antithrombotic drugs in the secondary prevention of ischemic stroke [60], means that ongoing and future trials of PFO closure will need to be very large. PFO closure may be more effective in subgroups with a stronger association with ischemic stroke (and therefore at highest risk of future cardioembolism), possibly in persons with very large PFO shunts [32] or PFO associated with atrial septal aneurysm [48].

## Supporting Information

Study characteristics of all included studies are given in full, along with referenced PRISMA flowchart detailing excluded studies. The MOOSE checklist is also submitted.

## Disclosure Statement

The authors have no conflict of interest to declare.

## Supplementary Material

Supplemental Table

## Figures and Tables

**Table 1:** Characteristics of included population-based and prospective studies

First author year	Design	Follow-years	Age (SD) years	Population	Exposure	Outcome	Confounders	Method of addressing confounding
*Migraine-stroke* Kurth, 2006 [21]	Cohort	10	55 (7.5)	Female health professionals	IHS structured interview	Ischemic stroke adjusted for known RF	Age, HTN, DM, chol, smoking, BMI, EtOH, menopause, HRT, HTN meds, chol meds, OCP, FHx MI	Cox regression
MacClellan, 2007 [22]	Case-control	NA	39 (5)	Cases: women identified through record linkage; controls: 10 year age-region-race matched population	IHS-based (MA only)	Cryptogenic	age, ethnicity, region, HTN, IHD, smoking, OCP	Logistic regression
*PFO-stroke* Di Tullio, 2007 [31]	Cohort	6.6^*^	69 (10)	Stroke free, population-based	TTE	Ischemic stroke adjusted for known RF	Age, sex, ethnicity, HTN, DM, smoking, chol, AF, aspirin use	Cox regression
Meissner, 2006 [30]	Cohort	5.1	67 (13)	Stroke/cardiac disease-free, population-based	TEE	Death, first or recurrent stroke/TIA	Age, sex, HTN, DM, IHD, smoking, chol, AF, ASA	Cox regression
Mas, 2001 [51]	Cohort	3.2	42 (6.5)	Cryptogenic stroke, hospital	TEE and TTE	Death or any recurrent cardio- or cerebrovascular event	Age, sex, HTN, DM, chol, smoking	Cox regression
De Castro, 2000 [61]	Cohort	2.6	50 (14)	Cryptogenic stroke/TIA, tertiary	TEE	Death or any recurrent stroke/TIA	Age, ‘classical vascular RF', treatment	Cox regression
Serena, 2008 [62]	Cohort	2.0	47 (15)	Cryptogenic stroke/TIA, tertiary	TCD then TEE	Any recurrent cerebrovascular event	Age, sex, HTN, DM, IHD, smoking, EtOH, migraine, ASA	Logistic regression
Feurer, 2010 [52]	Cohort	4.0	59 (15)	Ischemic stroke, hospital	TCD	Death or any recurrent cardio- or cerebrovascular event	Age, sex, HTN, DM, IHD, smoker, obesity, AF	Cox regression
Comess, 1994 [63]	Cohort	1.5	61 (10)	Ischemic stroke/TIA, hospital	TEE	Any recurrent stroke or TIA	Age, sex, HTN, DM, IHD, aspirin/warfarin use, carotid disease	Frequency matched
Petty, 2006 [33]	Case-control	NA	70 (10)	Cases: cryptogenic stroke, population; controls: random population	TEE	Cryptogenic stroke adjusted for known RF	Age, sex, HTN, IHD, LVF, smoking, AF, No. of contrast injections, ASA	Logistic regression
Sastry, 2006 [32]	Case-control	NA	33 (3)	Cases: ischemic stroke, hospital (2); controls: age-sex-GP-matched population	TCD validated with TEE in subgroup	Ischemic stroke adjusted for known RF	HTN, DM, smoking, chol	Conditional logistic regression
Roijer, 1997 [34]	Case-control	NA	69 (12)	Cases: stroke, hospital; controls: age-sex matched, population	TEE	Cryptogenic stroke	Age, sex, HTN, DM, IHD, AF	None
*PFO-migraine* Rundek, 2008 [45]	Cross-sectional	NA	69 (10)	Cases: stroke-free cohort, population-based; controls: cross-sectional	TTE		Age, sex, ethnicity, HTN, DM, chol, smoking	Logistic regression

HTN = Hypertension; DM = diabetes mellitus; IHD = ischemic heart disease; LVF = left ventricular failure; chol = hypercholesterolemia; OCP = oral contraceptive pill; ASA = atrial septal aneurysm; TIA = transient is-chemic attack; meds = medications; EtOH = alcohol intake; FHx MI = family history of myocardial infarction; HRT = hormone replacement therapy; AF = atrial fibrillation; RF = risk factor; MA = migraine with aura.

* Only mean follow-up reported.

Only prospective studies and/or population-based studies are presented here. Full characteristics of all considered studies are given in suppl. tables 3-5.
