# Supplementary material for: Patent Foramen Ovale, Ischemic Stroke and Migraine: Systematic Review and Stratified Meta-Analysis of Association Studies
Source: Neuroepidemiology. 2012 Oct 11;40(1):56–67. doi: 10.1159/000341924 (PMC3707011; doi:10.1159/000341924)
Supplement: Supplementary file 1 — Supplemental Table [file ned-0040-0056-s01.doc]

**Supplemental Methods**

| **MOOSE Checklist** | | | |
| --- | --- | --- | --- |
| **Criteria** | | **Brief description of how the criteria were handled in the review** | **Page** |
| **Reporting of background** | |  |  |
| √ | Problem definition | Previous meta-analyses of the association between migraine, patent foramen ovale (PFO) and ischemic stroke are inconsistent with data from randomized trials. We set out to conduct a systematic review and meta-analysis stratified by methodological quality to determine the strength of association. | 3 |
| √ | Hypothesis statement | Previous meta-analyses may have overestimated the associations between migraine, PFO and stroke because of pooling studies with methodological differences in study design. | 3 |
| √ | Description of study outcomes and exposures | Pairwise associations between migraine (IHS defined); PFO (contrast TEE, TTE, TCD during Valsalva); cryptogenic ischemic stroke (non-lacunar syndrome, carotid stenosis <50%, no cardiac arrhythmias or structural heart disease) or ischemic stroke (WHO criteria, supported by radiological findings) adjusted for known risk factors for ischemic stroke. | 4 |
| √ | Type of study designs used | Cohort studies; case-control; cross-sectional where adjustment for confounders. | 4-5 |
| √ | Study population | Any, except pregnant women, children and associations reported within family pedigrees. | 4-5 |
| **Reporting of search strategy should include** | |  |  |
| √ | Qualifications of searchers | Daniel Davis was trained in systematic methods of literature searching as part of postgraduate studies at University of Cambridge. John Gregson and Peter Willeit have similar training. Blossom Stephan, Rustam al-Shahi Salman and Carol Brayne are senior researchers with specific expertise in systematic reviews. |  |
| √ | Search strategy, including time period included in the synthesis and keywords | Search strategy (Pubmed) – see time periods below  Stroke:  (((("Stroke"[Mesh] OR ("Cerebrovascular Disorders"[Mesh] OR "Basal Ganglia Cerebrovascular Disease"[Mesh])) OR ("Brain Ischemia"[Mesh] OR "Cerebrovascular Disorders"[Mesh])) OR "Ischemic Attack, Transient"[Mesh]) OR ("Brain Infarction"[Mesh] OR "Cerebral Infarction"[Mesh])) OR (Stroke OR "Cerebrovascular Disorders" OR "Brain Ischemia" OR "Cerebrovascular Disease" OR "Ischemic Attack, Transient" OR "Brain Infarction" OR Cerebral Infarction) OR ("Brain Ischaemia" OR "Ischaemic Attack, Transient")))  PFO:  "Foramen Ovale" OR "Persistent Fetal Circulation Syndrome" OR "Embolism, Paradoxical" OR shunt OR interatrial shunt OR inter-atrial shunt  Migraine:  Migraine[MeSH]  Limits: Humans |  |
| √ | Effort to include all available studies | Studies in all languages were included  Authors were contacted where: abstracts were identified without a corresponding full publications | 4-5 |
| √ | Databases and registries searched | Medline (1950—), Embase (1980—), Science Citation Index (1950—), searched to 30^th^ November 2010. | 5 |
| √ | Search software used, name and version, including special features | Medline was accessed through Pubmed.  Embase was accessed through NHS Evidence Health Information Resources.  Science Citation Index is part of Web of Knowledge. | 5 |
| √ | Use of hand searching | The bibliography of all included studies and those of previous reviews on the subject were examined for further relevant studies. | 5 |
| √ | List of citations located and those excluded, including justifications | Studies were screened by title and abstract after de-duplication though first author surname, title and first page.  See table below: Sources of located citations  See PRISMA flowcharts for exclusions, along with reasons | 5 |
| √ | Method of addressing articles published in languages other than English | Italian and German articles were translated by Peter Willeit  Spanish articles were translated by John Gregson  Japanese articles were translated by Kanako Mori | 4 |
| √ | Method of handling abstracts or unpublished studies | Conference abstracts were retrieved and considered, but not included if insufficient information about methods reported. Authors contacted for full papers, if available. | 5 |
| √ | Description of any contact with authors | Authors of abstracts were contacted for full papers | 5 |
| **Reporting of methods should include** | |  |  |
| √ | Description of relevance or appropriateness of studies assembled for assessing the hypothesis to be tested | Tables of included studies (Tables S2—4) |  |
| √ | Rationale for the selection and coding of data | Studies were included or excluded as per criteria outlined above. | 5 |
| √ | Documentation of how data were classified and coded | Extracted data were used to classify methodologically similar groups of studies, based on the Newcastle-Ottawa Scale: population sampling; exposure / outcome definitions; confounders; methods for addressing confounding.  Study selection was performed by two researchers with disagreement resolved by consensus. Data were double extracted according to the piloted pro forma. | 5 |
| √ | Assessment of confounding | The methods for addressing confounding in each study are reported in Tables S2—4 |  |
| √ | Assessment of study quality, including blinding of quality assessors; stratification or regression on possible predictors of study results | Newcastle-Ottawa scale is a partially validated scale recommended by the Cochrane Collaboration.  Quality assessments were performed blind, with disagreement resolved by consensus. | 5 |
| √ | Assessment of heterogeneity | Heterogeneity of the studies was explored with *I*^2^ statistic. | 6 |
| √ | Description of statistical methods in sufficient detail to be replicated | We performed fixed effects meta-analysis using the metan command in Stata Version 10.1 | 6 |
| √ | Provision of appropriate tables and graphics | See Figures 2—4; Table 3; Tables S2—4. |  |
| **Reporting of results should include** | |  |  |
| √ | Graph summarising individual study estimates and overall estimate | Figures 2—4 |  |
| √ | Table giving descriptive information for each study included | Table 3 |  |
| √ | Results of sensitivity testing | None required, due to the low heterogeneity of important results |  |
| √ | Indication of statistical uncertainty of findings | 95% confidence intervals are presented with all summary estimates |  |
| **Reporting of discussion should include** | |  |  |
| √ | Quantitative assessment of bias | Bias was assessed on methodological grounds, guided by the Newcastle-Ottawa Scale, making further quantitative assessment of bias unnecessary. |  |
| √ | Justification for exclusion | All studies were excluded based on the pre-defined eligibility criteria. |  |
| √ | Assessment of quality of included studies | Quality was determined based on population sampling frames, guided by the Newcastle-Ottawa Scale. Eligibility criteria were strictly defined. |  |
| **Reporting of conclusions should include** | |  |  |
| √ | Consideration of alternative explanations for observed results | See Discussion | 9-10 |
| √ | Generalisation of the conclusions | See Discussion | 9-11 |
| √ | Guidelines for future research | See Discussion | 11 |
| √ | Disclosure of funding source | Daniel Davis is funded by a Wellcome research training fellowship. John Gregson is sponsored by a joint MRC / GlaxoSmithKline industrial CASE studentship. Peter Willeit is supported by a non-clinical PhD studentship from the British Heart Foundation. Blossom Stephan is funded by the Joint European Post-Doctoral Programme: The European Research Area in Ageing (ERA-AGE) Network FLARE Programme. Rustam Al-Shahi Salman was funded by a UK Medical Research Council clinician scientist fellowship.  Role of the Sponsors: No funding organisation or sponsor had any role in the design and conduct of the study, in the analysis and interpretation of the data, or in the preparation, review, or approval of the manuscript. No author has any other conflict of interest to declare. |  |

| **Sources of located citations** | | | | | | | | | |
| --- | --- | --- | --- | --- | --- | --- | --- | --- | --- |
|  | **Migraine-stroke** | | | **PFO-stroke** | | | **PFO-migraine** | | |
|  | Hits | Reviewed | Included | Hits | Reviewed | Included | Hits | Reviewed | Included |
| **Medline** | 2120 | 31 | 10 | 1036 | 62 | 21 | 196 | 23 | 5 |
| **SCI (unique)** | 1393 | 2 | 0 | 1065 | 7 | 1 | 149 | 2 | 0 |
| **Embase (unique)** | 913 | 5 | 0 | 637 | 2 | 0 | 177 | 4 | 0 |
| **Total** | 4426 | **10** | | 2738 | **22** | | 522 | **5** | |

**Supplemental Tables**

**Table S1.** Hierarchy of population sampling methods

| **Sampling strategy** | **Definition** | **Examples** | **Utility** |
| --- | --- | --- | --- |
| Population-based | All subgroups within geographical population included, unrestricted by demographic or clinical features | Randomized sampling from electoral register;  door-to-door recruitment; randomized telephone digit dialing | Lower risk of bias;  Results more generalizable |
| Community-convenience | Sampling from community setting, but with systematic element of selectivity | Volunteer participants;  Healthcare databases based on insurance reimbursements |  |
| Hospital-convenience | Sampling from hospital inpatient series, with systematic element of selectivity | Case-series of hospital patients matched to other hospital controls | Higher risk of bias;  Results less generalizable |

**Table S2.** Detailed reasons for exclusion of studies considered in previous meta-analyses

| **Study** | **Comments** |
| --- | --- |
| **Migraine—Stroke Systematic Review: Shurks 2009** [**^13^**](#_ENREF_13) | |
| Collab 1975 | Exposure: predates IHS classification, migraine assessed at interview, considered positive if reporting two or more: unilateral headache, throbbing quality, prodromal visual scintillation, vomiting, other symptoms. Not supported by validation studies. |
| Marini 1993 | Same source data as Carolei 1996 |
| Haapaniemi 1997 | Exposure: IHS criteria not used; migrainous infarction included.  Sampling: hospital cases only |
| Schwartz 1998 | Exposure: Self-reported migraine |
| Donaghy 2002 | Same source data as Chang 1999 |
| Pezzini 2007 | Population: only included stroke due to cervical artery dissection |
| Hall 2004 | Exposure: record-linkage of migraine report or triptan use (GPRD)  Outcome: record-linkage of stroke report (GPRD) |
| Velentgas 2004 | Exposure: record-linkage of migraine report or triptan use (Insurers database)  Outcome: record-linkage of stroke report (Insurers database) |
| Kurth 2007 | Exposure: self-reported migraine among male doctors (Physicians’ Health Study)  Outcome: self-reported stroke among male doctors |
| Stang 2005 | Outcome: stroke ascertained by computer algorithm and record-linkage |
| **Additional studies considered by Spector 2009** [**^53^**](#_ENREF_53) | |
| Lidegaard 2002 | Exposure: IHS criteria not used |
| Naess 2004 | Exposure: IHS criteria not used; self-reported migraine |
| Becker 2007 | Exposure: record-linkage of migraine report or triptan use (GPRD)  Outcome: record-linkage of stroke report (GPRD) |
| Buring 1995 | Exposure: self-reported migraine among male doctors (Physicians’ Health Study)  Outcome: self-reported stroke among male doctors |
| Merikangas 1997 | Outcome: self-reported physician diagnosis of stroke |
|  |  |
| **PFO—Stroke Systematic Review: Alsheikh 2009** [**^14^**](#_ENREF_14) | |
| Lechat 1988 | No adjustment or matching for possible confounders |
| Webster 1988 | No description of possible confounders in controls |
| Jeanrenaud 1990 | More complete data subsequently reported by Lausanne Stroke with Paradoxical Embolism Study Group |
| Hausmann 1992 | No adjustment or matching for possible confounders |
| De Belder 1992 | No adjustment or matching for possible confounders |
| Di Tullo 1993 | No non-stroke controls (case-series) |
| Ranoux 1993 | No non-stroke controls (case-series) |
| Homma 1994 | No adjustment or matching for possible confounders |
| Albers 1994 | No adjustment or matching for possible confounders |
| Klotzsch 1994 | No non-stroke controls (case-series) |
| Zahn 1995 | Peripheral and cerebral embolic events not considered separately |
| Schminke 1995 | No adjustment or matching for possible confounders |
| Yeung 1996 | No adjustment or matching for possible confounders |
| Steiner 1998 | No adjustment or matching for possible confounders |
| Kanda 1998 | Insufficient adjustment for cerebrovascular counfounders |
|  |  |
| **Migraine—PFO Systematic Review: Schwedt** [**^15^**](#_ENREF_15) | |
| Wilmshurst 2001 | Cross-sectional analysis of case-series of divers with decompression illness |
| Sztajzel 2002 | Cross-sectional analysis of case-series of cryptogenic stroke patients |
| Wilmshurst 2005 | No adjustment or matching for age or sex |
| Wilmshurst 2006 | Comparison of patients with stroke and migraine with stroke alone |
| Anzola 2006 | No adjustment or matching for possible confounders in case-series of patients with PFO |
| Kimmelstiel 2007 | Patient groups derived from disparate sources: stroke and nonstroke participants amalgamated |

| **Table S3.** Characteristics of included studies: Migraine – ischemic stroke | | | | | | | | | | | | | | | | | | | | |
| --- | --- | --- | --- | --- | --- | --- | --- | --- | --- | --- | --- | --- | --- | --- | --- | --- | --- | --- | --- | --- |
|  | Design | Median follow up (years) | Age (SD) (years) | Case definition and source | | Control source | Exposure ascertainment | Confounders | | | | | | | | | Method of addressing confounding | Outcomes in exposed | Outcomes in unexposed | Adjusted association  (95% CI) |
|  |  |  |  | Case source | Stroke definition |  |  | Age | Sex | HTN | DM | SM | AF | Chol | IHD | Others |  | Cases: Non cases | Cases: Non cases |  |
| Kurth 2006[^84^](#_ENREF_84) | Cohort | 10 | 55 (8) | Female health professionals | Ischemic stroke | Female health professionals | IHS structured interview | ●● | NA | ●● | ●● | ●● |  | ●● |  | BMI, EtOH, menopause, HRT, HTN meds, chol meds, OCP, FHx MI | Cox proportional hazards | 47 : 5125 | 204 : 22715 | HR 1•2 (0•9-1•7) |
|  |  |  |  |  |  |  |  |  |  |  |  |  |  |  |  |  |  | MA 18 : 1434 | NR | HR 1•9 (1•2-3•1) |
|  |  |  |  |  |  |  |  |  |  |  |  |  |  |  |  |  |  | MwoA 17 : 2176 | NR | HR 1•3 (0•8-2•1) |
| Schwaag 2003[^85^](#_ENREF_85) | Case-control | N/A | 35 (7) | Hospital | Primary cryptogenic stroke/TIA | Hospital staff and stroke free patients | IHS structured interview | ●● | ●● | ● | ● | ● |  | ● |  | OCP | Matching, Logistic regression | NR :42 | 20: 140 | OR 1•1 (0•5-2•4) |
| Carolei 1996[^86^](#_ENREF_86) | Case-control | N/A | 36 (7) | Hospital | Ischemic | Railway workers | IHS-based semi-structured interview | ●● | ●● | ●● | ●● | ●● |  | ●● |  | EtOH, BMI, OCP | Matching, conditional logistic regression | 46 : 262 | 54 : 537 | OR 1•9 (1•1-3•1) |
| Chang 1999[^87^](#_ENREF_87) | Case-control | N/A | 36 (6) | Female hospital patients | Ischemic stroke | Female hospital patients | IHS-based  questionnaire | ●● | ●● | ●● |  | ●● |  |  |  | FHx migraine, EtOH, social class | Matching, conditional logistic regression | 26 : 385 | 26 : NR | OR 3•5 (1•3-9•6) |
|  |  |  |  |  |  |  |  |  |  |  |  |  |  |  |  |  |  | MA 19 : 392 | 17 : NR | OR 3•8 (1•3-11) |
|  |  |  |  |  |  |  |  |  |  |  |  |  |  |  |  |  |  | MwoA 7 : 404 | 9 : NR | OR 3•0 (0•7-14) |
| Barinagarr-ementeria 1998[^88^](#_ENREF_88) | Ccase-control | N/A | 36 (6) | Hospital | Ischemic stroke | Stroke free hospital patients | IHS assessment | ● | ● | ● |  | ●● |  | ● |  | OCP | Frequency matching, Logistic regression | 20 : 110 | 13 : 109 | OR 1•3 (0•6-3•1) |
| Tzourio  1995[^89^](#_ENREF_89) | Case-control | N/A | 36 (7) | Hospital | Females Ischemic stroke | Female stroke free hospital inpatients | IHS assessment | ●● | NA | ●● |  | ●● |  |  |  | OCP | Frequency matching, Logistic regression | 43 : 29 | 52 : 121 | OR 3•5 (1•8-6•4) |
|  |  |  |  |  |  |  |  |  |  |  |  |  |  |  |  |  |  | MA 10 : 62 | 10 : 163 | OR 6•2 (2•1-18) |
|  |  |  |  |  |  |  |  |  |  |  |  |  |  |  |  |  |  | MwoA 33 : 39 | 42 : 131 | OR 3•0 (1•5-5•8) |
| Tzourio 1993[^90^](#_ENREF_90) | Case-control |  | 56 (14) | Hospital | Ischemic stroke | Stroke free hospital inpatients | IHS assessment | ●● | ●● | ●● |  |  |  |  |  |  | Matching, Logistic regression | 41 : 171 | 34 : 178 | OR 1•3 (0•8-2•3) |
|  |  |  |  |  |  |  |  |  |  |  |  |  |  |  |  |  |  | MA 9 : 203 | 7 : 205 | OR 1•3 (0•5-3•8) |
|  |  |  |  |  |  |  |  |  |  |  |  |  |  |  |  |  |  | MwoA 19 : 193 | 23 : 189 | OR 0•8 (0•4-1•5) |
| MacClellan 2007[^91^](#_ENREF_91) | Case-control | NA | 39 (5) | Females from hospital discharge records | Cryptogenic stroke | General population | IHS-based (MA only) | ●● | NA | ●● | ●● | ●● |  |  | ●● | Race, region, OCP | Logistic regression | MA 75 : 117 | 178 : 436 | OR 1•4 (0•9-2•0) |
| Camerlingo 2010[^92^](#_ENREF_92) | Case-control | N/A | 35 (7) | Hospital | Primary ischemic stroke | Partners and relatives of cases | IHS assessment | ●● | ●● | ●● | ● | ● |  |  |  | OCP | Matching, stratification, Logistic regression | MA 35 : 129 | 9: 155 | OR 5•1 (2•3-11)^b^ |
| Henrich 1989[^93^](#_ENREF_93) | Case-control | N/A | 57 (6) | Hospital | Ischemic |  | IHS structured interview | ●● | ●● | ●● | ●● | ●● |  |  | ● |  | Matching, Logistic regression | MA 11 : 78 | 9 : 169 | OR 2•6 (0•9-7•2) |
| Values refer to any past history of migraine, unless subtype is specified.  Confounders: ● variable considered, but not included in regression models; ●● variable matched or adjusted for in regression model. Other variables reported if adjusted for in regression model  Abbreviations: BMI=body mass; chol=hypercholesterolemia; CI=confidence interval; DM=diabetes mellitus; EtOH=alcohol use; FHx=family history; HR=hazard ratio; HTN=hypertension; IHD=ischemic heart disease; IHS=international headache society; LVF=left ventricular failure; MA=MA migraine with aura; MI=myocardial infarction; MwoA=migraine without aura; NR=Not reported; OCP=oral contraceptive pill; OR=odds ratio; PFO=patent foramen ovale.  ^a^ unadjusted, with adjusted estimate reported p <0.01 ^b^ subgroup analysis of MA in women only | | | | | | | | | | | | | | | | | | | | |

| **Table S4.** Characteristics of included studies: PFO – ischemic stroke | | | | | | | | | | | | | | | | | | | | | | |
| --- | --- | --- | --- | --- | --- | --- | --- | --- | --- | --- | --- | --- | --- | --- | --- | --- | --- | --- | --- | --- | --- | --- |
|  | Design | Median follow up (years) | Age (SD) (years) | Case definition and source | | Control definition | Exposure ascertainment | Confounders | | | | | | | | | | | Method of addressing confounding | Outcomes in exposed | Outcomes in unexposed | Adjusted association  (95% CI) |
|  |  |  |  | Case source | Stroke definition |  |  | Age | Sex | HTN | DM | SM | | AF | | Chol | IHD | Others |  | Cases: Non cases | Cases: Non cases |  |
| Meissner 2006[^94^](#_ENREF_94) | Cohort | 5.1 | 67 (13) | General population, stroke/cardiac disease free | Stroke/TIA | General population | TEE | ●● | ●● | ● | ● | ● | ●● | |  | | ●● | ASA | Cox proportional hazards | 12 : 140 | 29 : 437 | HR 1•5 (0•7-2•9) |
| Di Tulilo 2007[^95^](#_ENREF_95) | Cohort | 6•6† | 69 (1) | General population, stroke free | Ischemic stroke | General population | TTE | ●● | ●● | ●● | ●● | ●● | ●● | | ●● | |  |  | Cox proportional hazards | 12 : 164 | 56 : 936 | HR 1•6 (0•9-3•1) |
| Mas 2001[^96^](#_ENREF_96) | Cohort | 3•2 | 42 (7) | Hospital patients with previous cryptogenic stroke | Recurrent stroke/TIA | Hospital patients with previous cryptogenic stroke | TEE & TTE | ●● | ●● | ●● | ●● | ●● |  | | ●● | |  |  | Cox proportional hazards | 13 : 216 | 16 : 304 | HR 1•3 (0•6-2•9) |
| De Castro[^97^](#_ENREF_97) | Cohort | 2•6 | 50 (14) | Hospital patients with previous cryptogenic stroke / TIA |  |  | TEE | ●● |  |  |  |  |  | |  | |  | ‘vascular risk factors and treatment’ | Cox proportional hazards | 8 : 74 | 18 : 86 | RR 0•5 (0•2-1•1)^a^ |
| Serena 2008[^98^](#_ENREF_98) | Cohort | 2 | 47 (15) | Hospital patients with previous stroke/TIA | Recurrent stroke/TIA | Hospital patients with previous stroke/TIA | TCD & TEE | ●● | ● | ● | ● | ● |  | | ● | | ● | ‘vascular risk factors’, index stroke subtype | Logistic regression | 16: 297 | 12 : 189 | OR 0•9 (0•4-2•4) |
| Feurer 2010[^99^](#_ENREF_99) | Cohort | 4 | 59 (15) | Hospital patients with previous cerebral ischaemia | Recurrent cerebral ischemia | Hospital patients with previous cerebral ischaemia | TCD | ●● | ●● | ●● | ●● |  | ●● | |  | | ●● | Index stroke subtype | Cox proportional hazards | 10 : 254 | 32 : 509 | HR 0•8 (0•4-1•7) |
| Comess 1994[^100^](#_ENREF_100) | Cohort | 1•5 | 61 (17-85) | Hospital | Recurrent stroke or TIA | Hospital | TEE | ● | ● | ● | ● |  |  | |  | | ● |  | Frequency matched | 14 per 100 PY | 6•9 per 100 PY | RR 2•1 (0•9-5•0) |
| Cujec 1999[^101^](#_ENREF_101) | Cohort* | 3•8 | 41 (11) | Hospital patients with previous cryptogenic stroke/TIA | Cryptogenic stroke/ TIA | Hospital patients with previous cryptogenic stroke/TIA | TEE | ●● | ●● | ●● | ●● | ●● |  | | ●● | |  | OCP, DVT, migraine, valsava at ictus | Cox proportional hazards | 14 : 52 | 6 : 38 | RR 5•3 (1•6-18) |
| Roijer 1997[^102^](#_ENREF_102) | Case-control | N/A | 69 (12) | Stroke cases from hospital catchment area | Cryptogenic stroke | General population | TEE | ●● | ●● |  |  |  |  | |  | |  |  | Matching (incomplete) | 17 : 50 | 15 : 43 | OR 1•0 (0•4-2•2)^c^ |
| Sastry 2006[^103^](#_ENREF_103) | Case-control | N/A | 33 (17-39) *** | Hospital patients with previous ischemic stroke or MI | Ischemic stroke | General population | TCD^g^ | ●● | ●● | ●● | ●● | ●● |  | | ●● | |  |  | Conditional logistic regression | Any PFO 43 : 58 | Any PFO 38 : 63 | OR 1•3 (0•7-2•4) |
|  |  |  |  |  |  |  |  |  |  |  |  |  |  |  |  |  |  |  |  | Large PFO 24 : 77 | Large PFO 12 : 89 | OR 2•8 (1•2-6•5) |
| Petty 2006[^104^](#_ENREF_104) | Case-control | N/A | 70 (10) | Cryptogenic stroke patients from general population | Cryptogenic stroke | General population | TEE | ●● | ●● | ●● |  | ●● | ●● | |  | | ●● | no• of contrast injections, ASA, aortic stenosis | Logistic regression | Any PFO 33 : 100 | Any PFO 128 : 391 | OR 1•3 (0•8-2•2) |
|  |  |  |  |  |  |  |  |  |  |  |  |  |  |  |  |  |  |  |  | Large PFO 22 : 111 | Large PFO 108 : 411 | OR 0•9 (0•5-1•6) |
| Jones 1994[^105^](#_ENREF_105) | Case-control | N/A | 66 (13) | Hospital stroke patients | Cryptogenic stroke | Healthy volunteers |  | ● | ● |  |  |  |  | |  | |  |  | Frequency matching | 14 : 57 | 31 : 171 | OR 1•4 (0•7-2•7) |
| Serena 1998[^106^](#_ENREF_106) | Case-control | N/A | 64 (12) | Hospital stroke patients | Ischemic/ Cryptogenic stroke | Relatives of cases | TCD/TEE | ●● | ●● | ●● | ●● | ●● | ●● | | ●● | | ●● |  | Logistic regression | Any PFO 30 : 25 | Any PFO 32 : 68 | OR 2•6 (1•3-5•0)^d^ |
|  |  |  |  |  |  |  |  |  |  |  |  |  |  |  |  |  |  |  |  | Large PFO 24 : 31 | Large PFO 21 : 79 | OR 2•9 (1•4-6•0)^d^ |
| Force 2008[^107^](#_ENREF_107) | Cross-sectional | N/A | 71 (9) | Hospital TIA/ stroke patients | Cryptogenic stroke | Hospital TIA/stroke patients with known etiology | TEE | ●● | ● | ●● | ● | ● |  | |  | | ● |  | Logistic regression | 17 : 45 | 4 : 66 | OR 3•8 (0•7-21) |
| di Tullio 1992[^108^](#_ENREF_108) | Cross-sectional | N/A | 62 (15) | Hospital stroke patients | Cryptogenic stroke | Hospital stroke patients with known etiology | TTE | ●● | ● | ●● | ●● | ● |  | |  | |  |  | Logistic regression | 19:26 | 7 : 94 | OR 7•2 (2•4-22) |
| Cabanes 1993[^109^](#_ENREF_109) | Case-control | N/A | 39 (10) | Hospital stroke patients | Cryptogenic stroke | Hospital patients referred for non stroke related TEE | TEE | ● | ● | ● | ● | ● |  | | ● | |  | MVP, ASA | Logistic regression | 36 : 28 | 9 : 41 | OR 3•9 (1•5-9•9) |
| Negrao 2007[^110^](#_ENREF_110) | Cross-sectional | N/A | 34 (9) | Hospital stroke patients | Cryptogenic stroke | Hospital stroke patients with known etiology | TCD & TEE | ● | ● | ● | ● | ● |  | | ● | |  | infarct number & territory | Logistic regression | 35 : 53 | 12 : 68 | OR 3•3 (1•5-7•4) |
| Cerrato 2002[^111^](#_ENREF_111) | Case-control | N/A | 49^**^ | Hospital stroke patients | Cryptogenic stroke | Hospital lacunar stroke/TIA patients | TEE | ●● | ●● | ●● | ●● | ● |  | |  | |  | ASA | Stratification, logistic regression | 43 : 63 | 12 : 57 | OR 2•2 (1•1-4•9) |
| Schuchlenz 2000[^112^](#_ENREF_112) | Case-control | N/A | 43 (12) | Hospital stroke patients | Cryptogenic stroke | Referred for non stroke related TEE | TEE | ●● | ●● |  |  |  |  | |  | |  |  | Logistic regression | 1-4mm: 24 : 22 | NR | OR 1•5 (0•8-2•7)^e^ |
|  |  |  |  |  |  |  |  |  |  |  |  |  |  |  |  |  |  |  |  | > 4mm: 13 : 33 | NR | OR 12 (3•3-44)^e^ |
| Handke 2007[^113^](#_ENREF_113) | Cross-sectional | N/A | 62 (13) | Hospital stroke patients | Cryptogenic stroke | Hospital stroke patients with known etiology | TEE | ●● | ● | ●● | ● | ● |  | | ● | | ●● | aortic plaque thickness | Logistic regression | 77 : 150 | 34 : 242 | OR 3•1 (2•0-5•1) |
| Chen 1991[^114^](#_ENREF_114) | Case-control | N/A | 39 (8) | Cerebral/retinal ischemia hospital patients | Cryptogenic stroke | Vascular disease free hospital patients | TTE /TEE | ●● | ●● |  |  |  |  | |  | |  |  | Matching | TTE 4 : 30 | TTE 1 : 33 | OR 4•4 (0•5-42) |
|  |  |  |  |  |  |  |  |  |  |  |  |  |  |  |  |  |  |  |  | TEE 7 : 27 | TEE 15 : 19 | OR 3•1 (1•0-8•9) |
| Mattioli 2003[^115^](#_ENREF_115) | Case-control | N/A | 66 (21) | Hospital stroke/TIA patients | Cryptogenic stroke | Referred for non stroke related TEE• Aged <55 | TTE & TEE | ●● | ●● | ● |  | ● |  | |  | | ● | ASA | Logistic regression, matching | TTE 56 : 189 | TTE 24 : 221 | OR 2•7 (1•6-4•6)^d^ |
|  |  |  |  |  |  |  |  |  |  |  |  |  |  |  |  |  |  |  |  | TEE 50 : 40 | TEE 25 : 65 | OR 3•3 (1•7-6•0)^d^ |
| Values refer to any past history of migraine, unless subtype is specified.  Confounders: ● variable considered, but not included in regression models; ●● variable matched or adjusted for in regression model. Other variables reported if adjusted for in regression model  Abbreviations: ASA=atrial septal aneurysm; chol=hypercholesterolaemia; CI=confidence interval; DM=diabetes mellitus; HR=hazard ratio; HTN=hypertension; IHD=ischemic heart disease; LVF=left ventricular failure; MVP=mitral valve prolapse; OCP=oral contraceptive pill; OR=odds ratio; PFO=patent foramen ovale; PVD=peripheral vascular disease; PY=person-years; RR=rate ratio; TCD=transcranial Doppler; TEE transesophageal echocardiogram; TTE transthoracic echocardiogram.  * Only mean follow-up reported ** no standard deviation (SD) reported *** Mean (range)  a Crude rate ratio, with no change with adjustment reported, results of Cox regression reported as not significant b Retrospective c Crude OR, more AF in cryptogenic participants d Crude OR, adjusted reported as p < 0.01  e OR for one stroke only f Defined as nonlacunar g TEE validation performed in subgroup | | | | | | | | | | | | | | | | | | | | | | |

| **Tables S5.** Characteristics of included studies: PFO – Migraine | | | | | | | | | | | | | | | | | | | |
| --- | --- | --- | --- | --- | --- | --- | --- | --- | --- | --- | --- | --- | --- | --- | --- | --- | --- | --- | --- |
|  | Design | Age (SD) (years) | Population source | | Exposure ascertainment | Migraine definition | Confounders | | | | | | | | | Method of addressing confounding | Outcomes in exposed | Outcomes in unexposed | Adjusted association  (95% CI) |
|  |  |  | Cases | Controls |  |  | Age | Sex | HTN | DM | SM | AF | Chol | IHD | Others |  | Cases: Non cases | Cases: Non cases |  |
| Rundek 2008[^116^](#_ENREF_116) | Cross-sectional | 69 (10) | General population stroke free | | TTE | IHS-based questionnaire | ●● | ●● | ●● | ●● | ●● |  | ●● |  | Race | Logistic regression | 26 : 178 | 138 : 923 | OR 1•0 (0•6-1•6) |
| Garg 2010[^117^](#_ENREF_117) | Cross-sectional | 41 (12) | Headache center | Healthy volunTEErs | TTE and TCD | Neurology interview, IHS | ●● | ●● |  |  |  |  |  |  | Race, FHx stroke, FHx migraine | Matching, conditional logistic regression | 38 : 106 | 37 : 107 | OR 1•0 (0•6-1•7) |
| Tatlidede 2007[^118^](#_ENREF_118) | Cross-sectional | 38 (10) | Outpatient clinic | NR | TTE | Neurology interview, IHS | ● | ● |  |  |  |  |  |  |  | Frequency matching | 28 : 25 | 6 : 21 | OR 3•9 (1•4-11) |
| Anzola 2000[^60^](#_ENREF_60) | Cross-sectional | 33 (12) | Outpatient clinic | Hospital staff and patients | TCD | Neurology interview (MA), IHS | ●● | ●● |  |  |  |  |  |  |  | Logistic regression | 72:83 | 14:60 | OR 3•5 (1•8-6•8) |
| Schwerzmann 2005[^119^](#_ENREF_119) | Cross-sectional | 36* | Migraine clinic | Hospital staff & divers |  | Neurology interview (MA), IHS | ● | ● |  |  |  |  |  |  |  | Frequency matching (incompletely) | 44:49 | 16 : 77 | OR 4•6 (2•0-10•6) |
| Values refer to any past history of migraine, unless subtype is specified.  Confounders: ● variable considered, but not included in regression models; ●● variable adjusted for in regression model. Other variables reported if adjusted for in regression model  Abbreviations: chol=hypercholesterolaemia; CI=confidence interval; DM=diabetes mellitus; FHx=family history; HR=hazard ratio; HTN=hypertension; IHD=ischemic heart disease; IHS=international headache society; MA=MA migraine with aura; MwoA=migraine without aura; NR=Not reported; OCP=oral contraceptive pill; OR=odds ratio; PFO=patent foramen ovale  * No Standard Deviation (SD) reported | | | | | | | | | | | | | | | | | | | |

**Figure S1:** PRISMA Flow Diagram. Migraine—Stroke

**Screening**

**Included**

**Eligibility**

**Identification**

Records identified through database searching
(n = 8983)

Additional records identified through other sources
(n = 0)

Records after duplicates removed
(n = 4426)

Records screened
(n = 4426)

Records excluded
(n = 4388)

Full-text articles assessed for eligibility
(n = 31)

Full-text articles excluded, with reasons
(n = 21)

Inadequate definition of exposure (n = 7)[^7-15^](#_ENREF_7)

Inadequate definition of outcome (n = 5)[^20-24^](#_ENREF_20)

No case-level selection or adjustment for confounders (n = 2) [^32-34^](#_ENREF_32)

Only considered cervical artery dissection (n = 1)[^75^](#_ENREF_75)

Multiple reports describing same study (n = 4)

Studies included in qualitative synthesis
(n = 10)

Studies included in quantitative synthesis (meta-analysis)
(n = 9)

**Figure S2:** PRISMA Flow Diagram. PFO—Stroke

**Screening**

**Included**

**Eligibility**

**Identification**

Records identified through database searching
(n =4803)

Additional records identified through other sources
(n = 4)

Records after duplicates removed
(n = 2738)

Records screened
(n = 2738)

Records excluded
(n = 2679)

Full-text articles assessed for eligibility
(n = 59)

Full-text articles excluded, with reasons
(n = 38 )

Incorrect population (n = 1)[^6^](#_ENREF_6)

Inadequate definition of exposure (n = 3) [^16-18^](#_ENREF_16)

Inadequate definition of outcome (n = 3) [^29-31^](#_ENREF_29)

No case-level selection or adjustment for confounders (n = 17) [^37-53^](#_ENREF_37)

No analysis of outcome by exposure (n = 1)[^61^](#_ENREF_61)

Multiple reports describing same study (n = 6)

Only followed up PFO patients (n = 5)[^68-72^](#_ENREF_68)

Only reported trial data (n = 2)

Studies included in qualitative synthesis
(n = 21)

Studies included in quantitative synthesis (meta-analysis)
(n = 19)

**Figure S3:** PRISMA Flow-diagram. Migraine—PFO

**Identification**

Records identified through database searching
(n = 1293 )

Additional records identified through other sources
(n = 0)

Records after duplicates removed
(n = 522 )

**Screening**

**Included**

**Eligibility**

Records screened
(n = 522 )

Records excluded
(n = 493)

Full-text articles assessed for eligibility
(n = 26 )

Full-text articles excluded, with reasons
(n = 21 )

Incorrect populations (n =5)[^1-5^](#_ENREF_1)

Inadequate definition of outcome (n = 1)[^19^](#_ENREF_19)

Only abstract information (n = 4)[^25-28^](#_ENREF_25)

Reported associations in family pedigrees (n=3)

No case-level selection or adjustment for confounders (n = 5)[^54-58^](#_ENREF_54)

Multiple reports describing same study (n = 2)

Studies included in qualitative synthesis
(n = 5 )

Studies included in quantitative synthesis (meta-analysis)
(n = 3 )

**Supplementary References**

**1.** Truong T, Slavin L, Kashani R, et al. Prevalence of migraine headaches in patients with congenital heart disease. *Am J Cardiol.* Feb 1 2008;101(3):396-400.

**2.** Angeli S, Carrera P, Del Sette M, et al. Very high prevalence of right-to-left shunt on transcranial Doppler in an Italian family with cerebral autosomal dominant angiopathy with subcortical infarcts and leukoencephalopathy. *Eur Neurol.* 2001;46(4):198-201.

**3.** Milhaud D, Bogousslavsky J, van Melle G, Liot P. Ischemic stroke and active migraine. *Neurology.* Nov 27 2001;57(10):1805-1811.

**4.** Carod-Artal FJ, Ribeiro LD, Braga H, Kummer W, Mesquita HM, Vargas AP. Prevalence of patent foramen ovale in migraine patients with and without aura compared with stroke patients. A transcranial Doppler study. *Cephalalgia.* 2006;26(8):934-939.

**5.** Martin Balbuena S, Fuentes B, Lara M, Ortega-Casarrubios MA, Martinez P, Diez-Tejedor E. [Migraine as predictive factor of the presence of atrial septum aneurysm in patients with stroke and patent foramen ovale]. *Neurologia.* Apr 2009;24(3):160-164.

**6.** STEEnblik MH, Mineau GP, Pimentel R, Michaels AD. Population-based assessment of familial inheritance and neurologic comorbidities among patients with an isolated atrial septal defect. *Congenit Heart Dis.* Nov 2009;4(6):459-463.

**7.** Kurth T, Gaziano JM, Cook NR, et al. Migraine and risk of cardiovascular disease in men. *Arch Intern Med.* Apr 23 2007;167(8):795-801.

**8.** Nightingale AL, Farmer RD. Ischemic stroke in young women: a nested case-control study using the UK General Practice Research Database. *Stroke.* Jul 2004;35(7):1574-1578.

**9.** Hall GC, Brown MM, Mo J, MacRae KD. Triptans in migraine: the risks of stroke, cardiovascular disease, and death in practice. *Neurology.* Feb 24 2004;62(4):563-568.

**10.** Lidegaard O, Kreiner S. Contraceptives and cerebral thrombosis: a five-year national case-control study. *Contraception.* Mar 2002;65(3):197-205.

**11.** Schwartz SM, Petitti DB, Siscovick DS, et al. Stroke and use of low-dose oral contraceptives in young women: a pooled analysis of two US studies. *Stroke.* Nov 1998;29(11):2277-2284.

**12.** Oral contraceptives and stroke in young women. Associated risk factors. *JAMA.* Feb 17 1975;231(7):718-722.

**13.** Haapaniemi H, Hillbom M, Juvela S. Lifestyle-associated risk factors for acute brain infarction among persons of working age. *Stroke.* Jan 1997;28(1):26-30.

**14.** Naess H, Nyland HI, Thomassen L, Aarseth J, Myhr KM. Long-term outcome of cerebral infarction in young adults. *Acta Neurol Scand.* Aug 2004;110(2):107-112.

**15.** Kimmelstiel C, Gange C, Thaler D. Is patent foramen ovale closure effective in reducing migraine symptoms? A controlled study. *Catheter Cardiovasc Interv.* Apr 1 2007;69(5):740-746.

**16.** Bi Q, Wang L, Li X, Song Z. Risk factors and treatment of stroke in Chinese young adults. *Neurol Res.* May 2010;32(4):366-370.

**17.** Agmon Y, Khandheria BK, Meissner I, et al. Frequency of atrial septal aneurysms in patients with cerebral ischemic events. *Circulation.* Apr 20 1999;99(15):1942-1944.

**18.** Sugaya T, Yasu T, Fujii M, et al. Effect of atrial septal aneurysm on risk of cerebrovascular events in Japanese patients. *Int J Cardiol.* Feb 2004;93(2-3):253-256.

**19.** Pezzini A, Lodigiani C, Patella R, et al. Predictive value of cardiovascular risk factors, patent foramen ovale, and prothrombotic genotypes on migraine subtypes in young adults with ischemic stroke. *Pathophysiology of Haemostasis and Thrombosis.* 2010;37:A64.

**20.** Stang PE, Carson AP, Rose KM, et al. Headache, cerebrovascular symptoms, and stroke: the Atherosclerosis Risk in Communities Study. *Neurology.* May 10 2005;64(9):1573-1577.

**21.** Scher AI, Terwindt GM, Picavet HS, Verschuren WM, Ferrari MD, Launer LJ. Cardiovascular risk factors and migraine: the GEM population-based study. *Neurology.* Feb 22 2005;64(4):614-620.

**22.** Mitchell P, Wang JJ, Currie J, Cumming RG, Smith W. Prevalence and vascular associations with migraine in older Australians. *Aust N Z J Med.* Oct 1998;28(5):627-632.

**23.** Velentgas P, Cole JA, Mo J, Sikes CR, Walker AM. Severe vascular events in migraine patients. *Headache.* Jul-Aug 2004;44(7):642-651.

**24.** Wijman CA, Wolf PA, Kase CS, Kelly-Hayes M, Beiser AS. Migrainous visual accompaniments are not rare in late life: the Framingham Study. *Stroke.* Aug 1998;29(8):1539-1543.

**25.** De Heij AH, Luermans JGLM, Thijs V, et al. Patent foramen ovale with atrial septal aneurysm is a strong independent predictor for migraine with aura: A large prospective observational study. *European Heart Journal.* 2010;31:865.

**26.** Koppen H, Palm-Meinders IH, Mess WH, et al. Population-based evidence for an association between migraine and right-to-left shunt (PFO). *Cephalalgia.* 2009;29(12):1352-1352.

**27.** Marcovitz PA, Tobin KJ, Cronin L. Patent Foramen ovalle is more common in migraine headache sufferers than in controls. *Journal of the American College of Cardiology.* 2003;41(6):466A-466A.

**28.** Angeli S, Del Sette M, Bruzzone GL, Focacci A, Finocchi C, Gandolfo C. Migraine and right/left shunt with transcranial doppler. *Rivista di Neurobiologia.* 1998;44(2):155-159.

**29.** Huber M, Curtius JM, Hojer C, Vogelsberg H. TRANSESOPHAGEAL ECHOCARDIOGRAPHY DOES NOT IMPROVE CLINICAL RISK-ESTIMATION IN RECURRENT EMBOLIC STROKE. *Cerebrovascular Diseases.* 1994;4(1):38-43.

**30.** Zahn R, Lehmkuhl S, Lotter R, Zander M, Senges J. CARDIAC SOURCES OF CEREBRAL ISCHEMIC EVENTS WITH SPECIAL REGARD TO A PATENT FORAMEN OVALE. *Herz Kreislauf.* 1995;27(9):279-284.

**31.** Webster MW, Chancellor AM, Smith HJ, et al. Patent foramen ovale in young stroke patients. *Lancet.* Jul 2 1988;2(8601):11-12.

**32.** Mosek A, Marom R, Korczyn AD, Bornstein N. A history of migraine is not a risk factor to develop an ischemic stroke in the elderly. *Headache.* Apr 2001;41(4):399-401.

**33.** Alvarez J, Matias-Guiu J, Sumalla J, et al. Ischemic stroke in young adults. I. Analysis of the etiological subgroups. *Acta Neurol Scand.* Jul 1989;80(1):28-34.

**34.** Vitebskiy S, Fox K, Hoit BD. Routine transesophageal echocardiography for the evaluation of cerebral emboli in elderly patients. *Echocardiography.* Oct 2005;22(9):770-774.

**35.** Facheris M, Eisendle A, Grazio D, et al. Familial clustering of migraine and patent foramen ovage: Pilot study and genetic perspectives in a population dsolate. *Neurology.* 2008;70(11):A300-A300.

**36.** Wilmshurst PT, Pearson MJ, Nightingale S, Walsh KP, Morrison WL. Inheritance of persistent foramen ovale and atrial septal defects and the relation to familial migraine with aura. *Heart.* Nov 2004;90(11):1315-1320.

**37.** Yeung M, Khan KA, Shuaib A. Transcranial Doppler ultrasonography in the detection of venous to arterial shunting in acute stroke and transient ischemic attacks. *J Neurol Neurosurg Psychiatry.* Nov 1996;61(5):445-449.

**38.** Fazlinezhad A, Azimi S, Azarpazhooh M, Khajedaluee M, Kashani MM. Patent foramen ovale in young adults with cryptogenic stroke or transient ischemic attack. *Journal of Tehran University Heart Center.* 2009;4(3):185-188.

**39.** Itoh T, Matsumoto M, Handa N, et al. Paradoxical embolism as a cause of ischemic stroke of uncertain etiology. A transcranial Doppler sonographic study. *Stroke.* Apr 1994;25(4):771-775.

**40.** Fox ER, Picard MH, Chow CM, Levine RA, Schwamm L, Kerr AJ. Interatrial septal mobility predicts larger shunts across patent foramen ovales: an analysis with transmitral Doppler scanning. *Am Heart J.* Apr 2003;145(4):730-736.

**41.** Hausmann D, Mugge A, Becht I, Daniel WG. Diagnosis of patent foramen ovale by transesophageal echocardiography and association with cerebral and peripheral embolic events. *Am J Cardiol.* Sep 1 1992;70(6):668-672.

**42.** Jeanrenaud X, Bogousslavsky J, Payot M, Regli F, Kappenberger L. [Patent foramen ovale and cerebral infarct in young patients]. *Schweiz Med Wochenschr.* Jun 2 1990;120(22):823-829.

**43.** Job FP, Ringelstein EB, Grafen Y, et al. Comparison of transcranial contrast Doppler sonography and transesophageal contrast echocardiography for the detection of patent foramen ovale in young stroke patients. *Am J Cardiol.* Aug 15 1994;74(4):381-384.

**44.** Kanda N, Yasaka M, Otsubo R, Nagatsuka K, Minematsu K, Yamaguchi T. [Right-to-left shunt and atrial septal aneurysm in stroke patients: a contrast transesophageal echocardiographic study]. *Rinsho Shinkeigaku.* Mar 1998;38(3):213-218.

**45.** Kucherer H, Ratz K, Junger E, et al. [Recognition of cardiac normal variants as the cause of cerebral ischemia: significance of transesophageal echocardiography]. *Z Kardiol.* Dec 1996;85(12):917-923.

**46.** Lechat P, Mas JL, Lascault G, et al. Prevalence of patent foramen ovale in patients with stroke. *N Engl J Med.* May 5 1988;318(18):1148-1152.

**47.** Mesa D, Ruiz M, Delgado M, et al. Prevalence of patent foramen ovale determined by transesophageal echocardiography in patients with cryptogenic stroke aged 55 years or older. Same as younger patients? *Rev Esp Cardiol.* Mar 2010;63(3):315-322.

**48.** Musolino R, La Spina P, Granata A, et al. Ischaemic stroke in young people: a prospective and long-term follow-up study. *Cerebrovasc Dis.* 2003;15(1-2):121-128.

**49.** Negrao EM, Brandi IV, Nunes SV, Beraldo PS. [Abnormalities of interatrial septum and ischemic stroke in young people]. *Arq Neuropsiquiatr.* Dec 2005;63(4):1047-1053.

**50.** Petty GW, Khandheria BK, Chu CP, Sicks JD, Whisnant JP. Patent foramen ovale in patients with cerebral infarction. A transesophageal echocardiographic study. *Arch Neurol.* Jul 1997;54(7):819-822.

**51.** Ranoux D, Cohen A, Cabanes L, Amarenco P, Bousser MG, Mas JL. Patent foramen ovale: is stroke due to paradoxical embolism? *Stroke.* Jan 1993;24(1):31-34.

**52.** Silva MT, Rodrigues R, Tress J, Victer R, Chamie F. [Patent foramen ovale in a cohort of young patients with cryptogenic ischemic stroke]. *Arq Neuropsiquiatr.* Jun 2005;63(2B):427-429.

**53.** Vella MA, Sulke AN, Rodrigues CA, McNabb WR, Lewis RR. Patent foramina ovale in elderly stroke patients. *Postgrad Med J.* Aug 1991;67(790):745-746.

**54.** Domitrz I, Mieszkowski J, Kaminska A. Relationship between migraine and patent foramen ovale: a study of 121 patients with migraine. *Headache.* Oct 2007;47(9):1311-1318.

**55.** Ferrarini G, Malferrari G, Zucco R, Gaddi O, Norina M, Pini LA. High prevalence of patent foramen ovale in migraine with aura. *J Headache Pain.* Apr 2005;6(2):71-76.

**56.** Wilmshurst P, Pearson M, Nightingale S. Re-evaluation of the relationship between migraine and persistent foramen ovale and other right-to-left shunts. *Clin Sci (Lond).* Apr 2005;108(4):365-367.

**57.** Sztajzel R, Genoud D, Roth S, Mermillod B, Le Floch-Rohr J. Patent foramen ovale, a possible cause of symptomatic migraine: a study of 74 patients with acute ischemic stroke. *Cerebrovasc Dis.* 2002;13(2):102-106.

**58.** Del Sette M, Angeli S, Leandri M, et al. Migraine with aura and right-to-left shunt on transcranial Doppler: a case-control study. *Cerebrovasc Dis.* Nov-Dec 1998;8(6):327-330.

**59.** Domitrz I, Mieszkowski J, Kwiecinski H. [The prevalence of patent foramen ovale in patients with migraine]. *Neurol Neurochir Pol.* Mar-Apr 2004;38(2):89-92.

**60.** Anzola GP, Magoni M, Guindani M, Rozzini L, Dalla Volta G. Potential source of cerebral embolism in migraine with aura: a transcranial Doppler study. *Neurology.* May 12 1999;52(8):1622-1625.

**61.** Kizer JR, Segal AZ, Silverstein RL, et al. Prothrombotic Disorders, Patent Foramen Ovale and Unexplained Cerebral Infarction: The THrombophilia in Cryptogenic StroKe (THICK) Study. *Circulation.* 2008;118(18):S757-S757.

**62.** Mattioli AV, Aquilina M, Oldani A, Longhini C, Mattioli G. Atrial septal aneurysm as a cardioembolic source in adult patients with stroke and normal carotid arteries. A multicentre study. *Eur Heart J.* Feb 2001;22(3):261-268.

**63.** Meissner I, Whisnant JP, Khandheria BK, et al. Prevalence of potential risk factors for stroke assessed by transesophageal echocardiography and carotid ultrasonography: the SPARC study. Stroke Prevention: Assessment of Risk in a Community. *Mayo Clin Proc.* Sep 1999;74(9):862-869.

**64.** Sastry S, Riding G, Taberner D, Cherry N, Heagerty A, McCollum C. The role of venous-to-arterial circulation shunts and thrombophilia in ischemic stroke in young adults. *European Heart Journal.* 2003;24:91-91.

**65.** Agmon Y, Khandheria BK, Meissner I, et al. Comparison of frequency of patent foramen ovale by transesophageal echocardiography in patients with cerebral ischemic events versus in subjects in the general population. *Am J Cardiol.* Aug 1 2001;88(3):330-332.

**66.** Poppert H, Morschhaeuser M, Feurer R, et al. Lack of association between right-to-left shunt and cerebral ischemia after adjustment for gender and age. *J Negat Results Biomed.* 2008;7:7.

**67.** Steiner MM, Di Tullio MR, Rundek T, et al. Patent foramen ovale size and embolic brain imaging findings among patients with ischemic stroke. *Stroke.* May 1998;29(5):944-948.

**68.** Cerrato P, Priano L, Imperiale D, et al. Recurrent cerebrovascular ischemic events in patients with interatrial septal abnormalities: a follow-up study. *Neurol Sci.* Feb 2006;26(6):411-418.

**69.** Lee JY, Song JK, Song JM, et al. Association between anatomic features of atrial septal abnormalities obtained by omni-plane transesophageal echocardiography and stroke recurrence in cryptogenic stroke patients with patent foramen ovale. *Am J Cardiol.* Jul 1 2010;106(1):129-134.

**70.** Mas JL, Zuber M. Recurrent cerebrovascular events in patients with patent foramen ovale, atrial septal aneurysm, or both and cryptogenic stroke or transient ischemic attack. French Study Group on Patent Foramen Ovale and Atrial Septal Aneurysm. *Am Heart J.* Nov 1995;130(5):1083-1088.

**71.** Nedeltchev K, Arnold M, Wahl A, et al. Outcome of patients with cryptogenic stroke and patent foramen ovale. *J Neurol Neurosurg Psychiatry.* Mar 2002;72(3):347-350.

**72.** Stone DA, Godard J, Corretti MC, et al. Patent foramen ovale: association between the degree of shunt by contrast transesophageal echocardiography and the risk of future ischemic neurologic events. *Am Heart J.* Jan 1996;131(1):158-161.

**73.** Homma S, Sacco RL, Di Tullio MR, Sciacca RR, Mohr JP. Effect of medical treatment in stroke patients with patent foramen ovale: patent foramen ovale in Cryptogenic Stroke Study. *Circulation.* Jun 4 2002;105(22):2625-2631.

**74.** Homma S, DiTullio MR, Sacco RL, Sciacca RR, Mohr JP. Age as a determinant of adverse events in medically treated cryptogenic stroke patients with patent foramen ovale. *Stroke.* Sep 2004;35(9):2145-2149.

**75.** Pezzini A, Grassi M, Del Zotto E, et al. Migraine mediates the influence of C677T MTHFR genotypes on ischemic stroke risk with a stroke-subtype effect. *Stroke.* Dec 2007;38(12):3145-3151.

**76.** Kurth T, Schurks M, Logroscino G, Gaziano JM, Buring JE. Migraine, vascular risk, and cardiovascular events in women: prospective cohort study. *BMJ.* 2008;337:a636.

**77.** Kurth T, Slomke MA, Kase CS, et al. Migraine, headache, and the risk of stroke in women: a prospective study. *Neurology.* Mar 22 2005;64(6):1020-1026.

**78.** Donaghy M, Chang CL, Poulter N. Duration, frequency, recency, and type of migraine and the risk of ischemic stroke in women of childbearing age. *J Neurol Neurosurg Psychiatry.* Dec 2002;73(6):747-750.

**79.** Marini C, Carolei A, Roberts RS, et al. Focal cerebral ischemia in young adults: a collaborative case-control study. The National Research Council Study Group. *Neuroepidemiology.* 1993;12(2):70-81.

**80.** De Reuck J, Paemeleire K, Van Maele G. Stroke in patients with migraine. *Neurol Neurochir Pol.* Mar-Apr 2010;44(2):118-122.

**81.** Ueno Y, Kimura K, Iguchi Y, Shibazaki K, Inoue T, Urabe T. Right-to-left shunt and lacunar stroke in patients without hypertension and diabetes. *Neurology.* Feb 13 2007;68(7):528-531.

**82.** de Belder MA, Tourikis L, Leech G, Camm AJ. Risk of patent foramen ovale for thromboembolic events in all age groups. *Am J Cardiol.* May 15 1992;69(16):1316-1320.

**83.** Wilmshurst PT. The persistent foramen ovale and migraine. *Rev Neurol (Paris).* Jul 2005;161(6-7):671-674.

**84.** Kurth T, Gaziano JM, Cook NR, Logroscino G, Diener HC, Buring JE. Migraine and risk of cardiovascular disease in women. *JAMA.* Jul 19 2006;296(3):283-291.

**85.** Schwaag S, Nabavi DG, Frese A, Husstedt IW, Evers S. The association between migraine and juvenile stroke: a case-control study. *Headache.* Feb 2003;43(2):90-95.

**86.** Carolei A, Marini C, De Matteis G. History of migraine and risk of cerebral ischaemia in young adults. The Italian National Research Council Study Group on Stroke in the Young. *Lancet.* Jun 1 1996;347(9014):1503-1506.

**87.** Chang CL, Donaghy M, Poulter N. Migraine and stroke in young women: case-control study. The World Health Organisation Collaborative Study of Cardiovascular Disease and Steroid Hormone Contraception. *BMJ.* Jan 2 1999;318(7175):13-18.

**88.** Barinagarrementeria F, Gonzalez-Duarte A, Miranda L, Cantu C. Cerebral infarction in young women: analysis of 130 cases. *Eur Neurol.* Nov 1998;40(4):228-233.

**89.** Tzourio C, Tehindrazanarivelo A, Iglesias S, et al. Case-control study of migraine and risk of ischemic stroke in young women. *BMJ.* Apr 1 1995;310(6983):830-833.

**90.** Tzourio C, Iglesias S, Hubert JB, et al. Migraine and risk of ischemic stroke: a case-control study. *BMJ.* Jul 31 1993;307(6899):289-292.

**91.** MacClellan LR, Giles W, Cole J, et al. Probable migraine with visual aura and risk of ischemic stroke: the stroke prevention in young women study. *Stroke.* Sep 2007;38(9):2438-2445.

**92.** Camerlingo M, Romorini A, Ferrante C, Valente L, Moschini L. Migraine and cerebral infarction in young people. *Neurol Sci.* Jun 2010;31(3):293-297.

**93.** Henrich JB, Horwitz RI. A controlled study of ischemic stroke risk in migraine patients. *J Clin Epidemiol.* 1989;42(8):773-780.

**94.** Meissner I, Khandheria BK, Heit JA, et al. Patent foramen ovale: innocent or guilty? Evidence from a prospective population-based study. *J Am Coll Cardiol.* Jan 17 2006;47(2):440-445.

**95.** Di Tullio MR, Sacco RL, Sciacca RR, Jin Z, Homma S. Patent foramen ovale and the risk of ischemic stroke in a multiethnic population. *J Am Coll Cardiol.* Feb 20 2007;49(7):797-802.

**96.** Mas JL, Arquizan C, Lamy C, et al. Recurrent cerebrovascular events associated with patent foramen ovale, atrial septal aneurysm, or both. *N Engl J Med.* Dec 13 2001;345(24):1740-1746.

**97.** De Castro S, Cartoni D, Fiorelli M, et al. Morphological and functional characteristics of patent foramen ovale and their embolic implications. *Stroke.* Oct 2000;31(10):2407-2413.

**98.** Serena J, Marti-Fabregas J, Santamarina E, et al. Recurrent stroke and massive right-to-left shunt: results from the prospective Spanish multicenter (CODICIA) study. *Stroke.* Dec 2008;39(12):3131-3136.

**99.** Feurer R, Sadikovic S, Sepp D, et al. Patent foramen ovale is not associated with an increased risk of stroke recurrence. *Eur J Neurol.* Nov 2010;17(11):1339-1345.

**100.** Comess KA, DeRook FA, Beach KW, Lytle NJ, Golby AJ, Albers GW. Transesophageal echocardiography and carotid ultrasound in patients with cerebral ischemia: prevalence of findings and recurrent stroke risk. *J Am Coll Cardiol.* Jun 1994;23(7):1598-1603.

**101.** Cujec B, Mainra R, Johnson DH. Prevention of recurrent cerebral ischemic events in patients with patent foramen ovale and cryptogenic strokes or transient ischemic attacks. *Can J Cardiol.* Jan 1999;15(1):57-64.

**102.** Roijer A, Lindgren A, Algotsson L, Norrving B, Olsson B, Eskilsson J. Cardiac changes in stroke patients and controls evaluated with transesophageal echocardiography. *Scand Cardiovasc J.* 1997;31(6):329-337.

**103.** Sastry S, Riding G, Morris J, et al. Young Adult Myocardial Infarction and Ischemic Stroke: the role of paradoxical embolism and thrombophilia (The YAMIS Study). *J Am Coll Cardiol.* Aug 15 2006;48(4):686-691.

**104.** Petty GW, Khandheria BK, Meissner I, et al. Population-based study of the relationship between patent foramen ovale and cerebrovascular ischemic events. *Mayo Clin Proc.* May 2006;81(5):602-608.

**105.** Jones EF, Calafiore P, Donnan GA, Tonkin AM. Evidence that patent foramen ovale is not a risk factor for cerebral ischemia in the elderly. *Am J Cardiol.* Sep 15 1994;74(6):596-599.

**106.** Serena J, Segura T, Perez-Ayuso MJ, Bassaganyas J, Molins A, Davalos A. The need to quantify right-to-left shunt in acute ischemic stroke: a case-control study. *Stroke.* Jul 1998;29(7):1322-1328.

**107.** Force M, Massabuau P, Larrue V. Prevalence of atrial septal abnormalities in older patients with cryptogenic ischemic stroke or transient ischemic attack. *Clin Neurol Neurosurg.* Sep 2008;110(8):779-783.

**108.** Di Tullio M, Sacco RL, Gopal A, Mohr JP, Homma S. Patent foramen ovale as a risk factor for cryptogenic stroke. *Ann Intern Med.* Sep 15 1992;117(6):461-465.

**109.** Cabanes L, Mas JL, Cohen A, et al. Atrial septal aneurysm and patent foramen ovale as risk factors for cryptogenic stroke in patients less than 55 years of age. A study using transesophageal echocardiography. *Stroke.* Dec 1993;24(12):1865-1873.

**110.** Negrao EM, Brandi IV, Nunes SV, Tavora DG, Nakayama M, Beraldo PS. Patent foramen ovale and ischemic stroke in young people: statistical association or causal relation? *Arq Bras Cardiol.* May 2007;88(5):514-520.

**111.** Cerrato P, Imperiale D, Priano L, et al. Transesophageal echocardiography in patients without arterial and major cardiac sources of embolism: difference between stroke subtypes. *Cerebrovasc Dis.* 2002;13(3):174-183.

**112.** Schuchlenz HW, Weihs W, Horner S, Quehenberger F. The association between the diameter of a patent foramen ovale and the risk of embolic cerebrovascular events. *Am J Med.* Oct 15 2000;109(6):456-462.

**113.** Handke M, Harloff A, Olschewski M, Hetzel A, Geibel A. Patent foramen ovale and cryptogenic stroke in older patients. *N Engl J Med.* Nov 29 2007;357(22):2262-2268.

**114.** Chen WJ, Lin SL, Cheng JJ, Lien WP. The frequency of patent foramen ovale in patients with ischemic stroke: a transesophageal echocardiographic study. *J Formos Med Assoc.* Aug 1991;90(8):744-748.

**115.** Mattioli AV, Bonetti L, Aquilina M, Oldani A, Longhini C, Mattioli G. Association between atrial septal aneurysm and patent foramen ovale in young patients with recent stroke and normal carotid arteries. *Cerebrovasc Dis.* 2003;15(1-2):4-10.

**116.** Rundek T, Elkind MS, Di Tullio MR, et al. Patent foramen ovale and migraine: a cross-sectional study from the Northern Manhattan Study (NOMAS). *Circulation.* Sep 30 2008;118(14):1419-1424.

**117.** Garg P, Servoss SJ, Wu JC, et al. Lack of association between migraine headache and patent foramen ovale: results of a case-control study. *Circulation.* Mar 30 2010;121(12):1406-1412.

**118.** Tatlidede AD, Oflazoglu B, Celik SE, Anadol U, Forta H. Prevalence of patent foramen ovale in patients with migraine. *Agri.* Oct 2007;19(4):39-42.

**119.** Schwerzmann M, Nedeltchev K, Lagger F, et al. Prevalence and size of directly detected patent foramen ovale in migraine with aura. *Neurology.* Nov 8 2005;65(9):1415-1418.
